# Prognostic Value of Circulating Cytokines in Chemorefractory Colorectal Cancer

**DOI:** 10.3390/cancers15245823

**Published:** 2023-12-13

**Authors:** Irene Assaf, Danai Fimereli, Geraldine Anthoine, Roberta Fazio, Valentina Daprà, Alessandro Audisio, Alina Bardiaux, Tugba Akin Telli, Michele Vanhooren, Rita Saude-Conde, Giacomo Bregni, Alain Hendlisz, Francesco Sclafani

**Affiliations:** 1 Department of Digestive Oncology, Institut Jules Bordet, The Brussels University Hospital, 1070 Brussels, Belgium; irene.assaf@hubruxelles.be (I.A.); roberta.fazio@hubruxelles.be (R.F.); valentina.dapra@hubruxelles.be (V.D.); alessandro.audisio@hubruxelles.be (A.A.); tugbaakintelli@gmail.com (T.A.T.); michele.vanhooren@uzleuven.be (M.V.); rita.saudeconde@hubruxelles.be (R.S.-C.); giacomo.bregni@gamil.com (G.B.); alain.hendlisz@hubruxelles.be (A.H.); 2 Breast Cancer Translational Laboratory, Institut Jules Bordet, The Brussels University Hospital, 1070 Brussels, Belgium; danai.fimereli@hubruxelles.be; 3 GI Cancer Laboratory, Institut Jules Bordet, The Brussels University Hospital, 1070 Brussels, Belgium; geraldine.anthoine@hubruxelles.be (G.A.); alina.bardiaux@ulb.be (A.B.); 4 Medical Oncology, Faculty of Medecine, Erasmus Campus, Université Libre de Bruxelles (ULB), 1070 Brussels, Belgium

**Keywords:** cytokines, IGFBP-1, IGFBP-2, colorectal cancer

## Abstract

**Simple Summary:**

The prognosis of metastatic colorectal cancer patients remains poor despite the increased number of active treatments. This particularly applies to patients with chemorefractory disease. Biomarkers that could help prognostication and management decisions in this setting are lacking. This is the study with the largest panel (*n* = 182) of circulating cytokines ever assessed in metastatic colorectal cancer. By analyzing 196 chemorefractory colorectal cancer patients with available plasma samples, we showed that high concentrations of IGFBP-1 and/or IGFBP-2 were associated with shorter overall survival. These results are consistent with the extensive literature supporting an association between the IGF pathway and colorectal cancer. Further research is needed to validate our findings in larger independent series, and to elucidate the biological mechanisms underlying the prognostic effect of these cytokines.

**Abstract:**

Circulating cytokines could be optimal biomarkers for prognostication and management decisions in colorectal cancer (CRC). Chemorefractory CRC patients with available plasma samples were included in this study. In the discovery cohort (*n* = 85), 182 circulating cytokines were tested with a semi-quantitative multiplex assay, and prognostic cytokines were analyzed in the validation cohort (*n* = 111) by ELISA. Overall survival (OS) was the primary outcome measure, with the false discovery rate (FDR) method (significance level of <0.01) being used to correct for multiple comparisons. Four cytokines were associated with OS in the discovery cohort: insulin-like growth factor-binding protein 1 (IGFBP-1) (HR 2.1 [95%CI: 1.58–2.79], FDR < 0.001), insulin-like growth factor-binding protein 2 (IGFBP-2) (HR 1.65 [95%CI: 1.28–2.13], FDR = 0.006), serum amyloid A (SAA) (HR 1.84 [95%CI: 1.39–2.43], FDR < 0.001), and angiotensin II (HR 1.65 [95%CI: 1.29–2.1], FDR = 0.006). Of these, IGFBP-1 (HR 2.70 [95%CI: 1.56–4.76], FDR = 0.007) and IGFBP-2 (HR 3.33 [95%CI: 1.64–6.67], FDR = 0.008) were confirmed to be independently associated with OS in the validation cohort. Patients with high concentrations of IGFBP-1 and/or IGFBP-2 had a median OS of 3.0 months as compared with 6.9 months for those with low concentrations of both cytokines (HR 2.44 [95%CI: 1.52–4.0], FDR = 0.002) Validation of circulating IGFBP-1 and IGFBP-2 as independent prognostic biomarkers for chemorefractory CRC in larger, independent series is warranted.

## 1. Introduction

Colorectal cancer (CRC) is the third most common cancer and the second leading cause of cancer-related deaths worldwide [1]. Overall, approximately 45% of patients have metastatic disease, which can be detected at the time of the initial diagnosis or can occur at a later stage due to failure of curative intent treatments. While the availability of novel therapeutics and the increased use of multimodality approaches have gradually led to better outcomes, the prognosis of patients with metastatic disease remains poor, with survival ranging from 12 to 42 months depending on the tumor molecular profile and treatments received [2].

Over the years, there has been a particular focus on the identification of biomarkers that could unveil the inherent tumor biology of CRC, reveal druggable therapeutic targets and ultimately guide treatment decisions. Among these, dMMR/MSI, *KRAS*, *NRAS*, *BRAF*, *HER-2*, and *NTRK* are tested in clinical practice to offer personalized treatment strategies, with some geographic variations based on local reimbursement and drug access [3,4,5,6,7]. Despite continuous efforts to capture the molecular granularity of this malignancy, it is still common to see patients who share similar molecular profiles having different responses to treatment and/or prognosis, and the search for novel biomarkers that could account for such biological diversity is ongoing.

Optimal treatment selection and delivery for chemorefractory patients are some of the key challenges in the management of metastatic CRC. On the one hand, the proportion of patients who are candidates for further treatment is limited, real-world data suggesting that <40% will eventually receive a 3^rd^ line of systemic treatment largely due to rapid deterioration of clinical conditions or early death [8,9]. On the other hand, the number of therapeutic options in this setting has recently increased, and now includes combination treatments, novel multi-kinase inhibitors, the rechallenge of agents used in earlier lines of treatment, and experimental drugs from clinical trials [10,11]. Nevertheless, robust biomarkers that could help to refine prognostication and inform the decision-making are lacking.

Liquid biopsy is a revolutionary tool in the oncology field. Blood represents an ideal compartment for the analysis of a number of cancer biomarkers. So far, however, the bulk of research has focused on circulating tumor DNA and its clinical applications, such as early cancer detection, minimal residual disease, the identification of therapeutic targets and the assessment of treatment response/resistance [12]. Cytokines are a broad group of proteins that act as key mediators of cell signaling. They are produced by a variety of cells and exert their functions via autocrine, paracrine or endocrine mechanisms [13]. While involved in a plethora of physiological processes and sometimes used for therapeutic purposes, cytokines can play an important role in cancer formation, development and progression [14]. Further to the dysregulation of their levels and/or signaling pathways, they have been reported to mediate, among others, intercellular communications within the tumor microenvironment, promote angiogenesis, epithelial–mesenchymal transition and tumor invasion, influence host immune response and tumor immune evasion, support cancer stem-cell-like cells, promote metastasis, and contribute to treatment resistance [14,15]. Many studies have been conducted to better understand the pathogenic role and prognostic/predictive value of cytokines across tumor types. Very little, however, is known about the potential role and clinical applications of circulating cytokines in chemorefractory CRC. Furthermore, the available studies are limited by the small number of cytokines analyzed.

With this in mind, we carried out an exploratory analysis of a broad number of circulating cytokines in a population of chemorefractory CRC patients who were managed at our institution within the context of prospective clinical trials.

## 2. Material and Methods

### 2.1. Study Design and Eligibility Criteria

This is a retrospective, single-center study conducted at the Institut Jules Bordet, Brussels (Belgium). Patients were eligible if they had the following: cytologically or histologically confirmed colorectal adenocarcinoma, inoperable locally advanced or metastatic disease, previously received ≥1 line of systemic treatment, a blood sample prospectively taken at baseline (i.e., before the start of a new line of systemic treatment) within the context of an academic clinical trial sponsored by the Institut Jules Bordet, and provided consent for future biomarker analyses.

The study population consisted of a discovery and a validation cohort. The discovery cohort included eligible patients from the CORIOLAN trial and CHRONOS & KAIROS study. The former investigated the association between the natural pace of tumor progression (as indicated by tumor metabolic changes on serial 18F-FDG PET scans) and survival in chemorefractory CRC [16]. Following a 14-day observation period without any active anti-cancer therapy, patients from this trial could be treated off-study based on local standards. The latter is an ongoing study aiming to build a large, clinically annotated, bio-repository for gastrointestinal cancers that could serve as a platform for the discovery and validation of clinically useful biomarkers, with a specific focus on liquid biopsy. Patients from this study were treated according to standard practice. The validation cohort included patients who were enrolled in RegARd-C. This was a single-arm phase II trial exploring early predictive biomarkers of resistance to regorafenib in chemorefractory CRC [17]. Before collecting the blood samples that were used for this study, patients from both the discovery and validation cohorts were treated with standard therapies, including fluoropyrimidines, irinotecan, oxaliplatin, monoclonal antibodies (bevacizumab, cetuximab and/or panitumumab) and, only for the discovery cohort, regorafenib.

Eight healthy volunteers (one male and one female for each of the age groups 21–30, 31–40, 41–50, and 51–60) were included in the study, and asked to donate blood samples that would be used to set the cut-off for each of the cytokines tested in the validation cohort.

### 2.2. Cytokine Analysis

For all eligible study patients, plasma aliquots were processed and stored at −80 °C according to our institutional guidelines.

A total of 182 cytokines in the discovery cohort were analyzed with the “Human L182 Array, Membrane” (RayBio), a commercially available, semi-quantitative, multiplex assay that allows testing the expression of 182 cytokines simultaneously, including the following: ACE/CD43, ACE-2, ACTH, ADFP, Adiponectin, Adipsin (Factor D), AgRP, AMPKa1, Amylin, Angiopoietin 1, Angiopoietin 2, angiotensinogen/angiotensin II, Ang-like Factor, ANGPTL1, ANGPTL2, ANGPTL3, ANGPTL4, Apelin Receptor, ApoB, ApoE, Axl, BDNF, bFGF, BMP-2, BMP-3, BMP-3b/GDF-10, BMP-4, BMP-5, BMP-6, BMP-7, BMP-8, BMP-15, BMPR-IA/ALK-3, BMPR-IB/ALK-6, BMPR-II, b-NGF, C3a des Arg, CART, CD137 (4-1BB), CD36, Clusterin, CNTF, C-peptide, CRP, Cystatin C, Dtk, EGF, EGFR, ENA-78, Endophin Beta, Epiregulin, E-selectin, ET-1 (Endothelin), FABP4, FAM3B, FAS/Apo-1, FGF-10, FGF-6, FSH, Galectin-1, Growth Hormone (Growth Hormone), Ghrelin, GITR, GITR Ligand, GLP-1, Glucagon, Glut1, Glut2, Glut3, Glut5, Glutathione peroxidase 1, Glutathione peroxidase 3, GRO alpha, HCC4, HGF, HSD-1, ICAM1, IFN-gamma, IGF-1, IGF-1 sR, IGFBP-1, IGFBP-2, IGFBP-3, IGF-2, IL-1 R1, IL-1 R4, IL-1a, IL-1b, IL-1 ra, IL-6, IL-6 sR, IL-8, IL-10, IL-11, IL-12, IL-25/IL-17E, INSL3, INSRR, Insulin, Insulin R (CD220), Leptin, Leptin R, LH (Luteinizing Hormone), LIF, LOX, Lymphotactin, MCP-1, MCP-3, M-CSF, MIF, MIP-1 alpha, MIP-1 beta, MIP-3b, MMP-2, MMP-9, MMP-11, MMP-19, MSHa, MSPa, Myostatin, NAIP, NeuroD1, Neurophilin-2, NGF R, NPY (Neuropeptide Y), Obestatin R (GPR-39), Orexin A, Orexin B, OSM, Osteocalcin, Osteonectin, Osteoprotegerin, PARC, PDGF, PDGF-AA, PDGF-AB, PDGF-C, PDGF-D, PEDF, Pentraxin-3, PPARg2/NRIC3, Pref-1, Prohibitin, Prolactin, PYY, RANTES, RBP4, RELMb, Resistin, S100, S100 A8 + A9, S100 A10, SAA, SDF-1, SEMA3A, Serotonin, Syndecan-3, TACE, TDAG51, TECK, TGF alpha, TGF-b, Thrombospondin 1, Thrombospondin 2, Thrombospondin 4, TIMP-1, TIMP-2, TIMP-3, TIMP-4, Tissue factor (CD142), TLR2, TLR4, TNF alpha, sTNFRI, sTNFRII, TSG-6, TSH, Vaspin, VCAM-1, VEGF-A, Visfatin/PBEF1, and XEDAR.

In short, following biotinylation of the primary amine groups of the plasma proteins, 50 μL of plasma were added into a membrane array pre-printed with capture antibodies, and incubated to allow for the interaction of target proteins. After incubation with HRP-conjugated streptavidin, signals were visualized using a chemiluminescence imaging system and quantified by densitometry. After subtracting the local background, data for each cytokine were normalized to the positive control signals. All analyses were carried out at the manufacturer central laboratory (Tebubio, Boechout, Belgium).

Cytokine analysis in healthy volunteers and the validation cohort was conducted using an enzyme-linked immunosorbent assay (ELISA) test (sandwich or competitive depending on the cytokine) according to the manufacturer’s instructions (Abcam, Cambridge, UK). Plasma samples from each patient were subjected to dilution steps, based on the anticipated (according to the literature) concentration ranges for each of the analyzed cytokines, as well as sensitivity and detection range of each assay (Supplementary Files, Table S1). A total of 100 µL of diluted plasma were added to pre-coated 96-well plates (Abcam, Cambridge, UK) and incubated. A biotinylated antibody was added and incubated. In accordance with the supplier’s instructions, washing and revealing steps were carried out. The mean absorbance was measured on the microplate reader FLUOstar OPTIMA (BMG Labtech, Ortenberg, Germany) at 450 nm. The relative optical density (OD)450 was calculated as *(OD450 of each well)—(OD450 of Zero well).* A standard curve (either a classic graph or a log–log graph, depending on the cytokine) was plotted in an excel file as the relative OD450 of each standard solution (*y* axis) versus the respective concentration of the standard solution (*x* axis). The best-fit straight line through the standard points was chosen (eliminating discordant points if necessary), and concentration was measured through the linear equation *y* = *ax* + *b*. All analyses were carried out in duplicates.

### 2.3. Study Objective and Statistical Considerations

The objective of the study was to assess the prognostic value of a large panel of circulating cytokines in patients with chemorefractory CRC. OS was the primary outcome measure, and it was measured from the collection of the baseline blood sample to death from any cause. Patients lost to follow up were censored at the date of the last contact.

All cytokine data from study patients and healthy volunteers were log-normalized. In the discovery cohort, principal component analysis to identify outliers was performed. Median values of cytokines were considered for dichotomization between patients with high and low cytokine levels. Univariable analyses were carried out, and to correct for multiple comparisons, a Benjamini–Hochberg multiple correction procedure with a false discovery rate (FDR) approach and a significance level of <0.01 was applied. Significant cytokines from the discovery cohort were selected for analysis in the validation cohort, where the highest concentration among healthy volunteers was used as cut-off. The Spearman rank correlation test was used to assess the relationship between cytokines. The Kaplan–Meier method, Cox proportional hazards models and log-rank tests were used to estimate OS, and to test for associations. For multivariable analyses in the validation cohort, *p* values were derived via an ANOVA test on nested logistic and Cox models, including all baseline clinico-pathological variables that were prognostic in univariable analyses with a *p*-value of <0.1. Baseline variables analyzed included age, sex, ECOG performance status (PS), tumor sidedness, *RAS/BRAF* status, hemoglobin, leukocytes, platelets, alkaline phosphatase (ALP), carcinoembryonic antigen, timing of metastases (synchronous vs. metachronous), time from CRC diagnosis to blood sample collection, number of sites of active disease, whole-body metabolically active tumor volume (WB-MATV), presence of liver metastases, presence of peritoneal metastases and prior history of metastasectomy. All statistical analyses and plots were implemented in R (v4.0.2).

### 2.4. Ethical Aspects

The study was conducted in accordance with the principles of Good Clinical Practice and the Declaration of Helsinki. Given that, as a result of their participation in other studies, all patients had already provided consent for future research analyses in collected samples, neither approval from an Ethics Committee nor study-specific consent was required.

## 3. Results

Eighty-seven patients were eligible and initially included in the discovery cohort, forty-seven from CORIOLAN and forty from CHRONOS & KAIROS. After running a principal component analysis, two clustering groups were identified, largely corresponding to the two source studies. Two outliers with low values across all cytokines, suggesting technical failure, were found and excluded from further analysis (Supplementary Files, Figure S1).

Baseline characteristics of the assessable 85 patients from the discovery cohort are reported in Table 1. The median number of prior lines of systemic therapy was two (range 1–7), the median time from diagnosis to the blood sample collection was 32.2 months (range 2.1–196.6), the time from last chemotherapy to blood sampling was >2 weeks, and the median number of metastatic sites was three (range 1–6). The median ECOG PS was one, and the majority of patients (75.3%) received at least one more line of systemic therapy after the blood sample collection. The median follow up was 7 months. The median OS was 6.8 months (range 0.4–34.2), with 14.1% of patients being alive at the time of the last contact.

**Figure 1:**
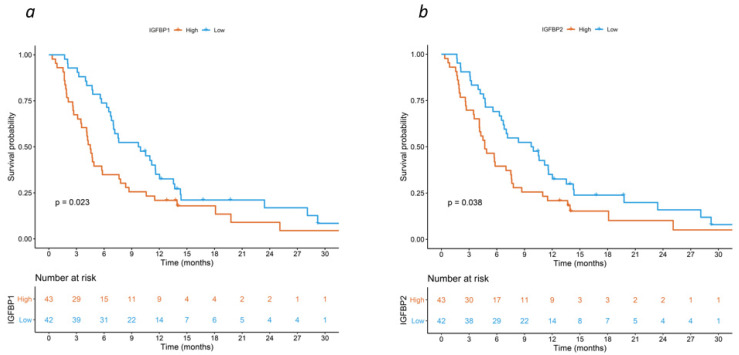
Kaplan–Meier curves for overall survival by expression of (**a**) IGFBP-1 and (**b**) IGFBP-2 in the discovery cohort.

The full results of the semi-quantitative, multiplex cytokine analysis are not shown, but are available upon reasonable request. After correction for the source study (i.e., CORIOLAN vs. CHRONOS & KAIROS), only 4 out of the 182 tested cytokines were found to be associated with OS, meeting the criteria for statistical significance at the FDR threshold of <0.01. These included insulin-like growth factor-binding protein 1 (IGFBP-1) (HR 2.1 [95%CI: 1.58–2.79], FDR < 0.001), insulin-like growth factor-binding protein 2 (IGFBP-2) (HR 1.65 [95%CI: 1.28–2.13], FDR = 0.006), serum amyloid A (SAA) (HR 1.84 [95%CI: 1.39–2.43], FDR < 0.001), and angiotensin II (HR 1.65 [95%CI: 1.29–2.1], FDR = 0.006). The corresponding survival curves are shown in Figure 1 and Figure S2 in the Supplementary Files.

The four cytokines found to be significant in the discovery cohort were then tested using ELISA in healthy volunteers. The highest concentrations were 94.1 ng/mL for IGFBP-1, 1458.2 ng/mL for IGFBP-2, 10,742.4 ng/mL for SAA, and 2.4 ng/mL for angiotensin II, and these were used as cut-off values for subsequent analyses in the validation cohort (Supplementary Files, Table S2).

The validation cohort consisted of 111 patients. Their baseline characteristics are reported in Table 1. The median number of prior lines of systemic therapy was two (range 1–7), the median time from diagnosis to the blood sample collection was 37.3 months (range 5.9–155.5), the time from last chemotherapy to blood sampling was >4 weeks, and the median number of metastatic sites was three (range 1–7). The median ECOG PS was one, and all patients received at least one more line of systemic therapy after the blood sample collection. The median follow up was 6 months. The median OS was 5.8 months (range 0–25), with 4.5% of patients being alive at the time of the last contact. Among all the baseline clinico-pathological factors analyzed in this cohort, ALP (HR 1.70 [95%CI: 0.98–2.96], *p* = 0.05) and WB-MATV (HR 2.56 [95%CI: 1.49–4.39], *p* = 0.001) were independent prognostic variables.

**Figure 2:**
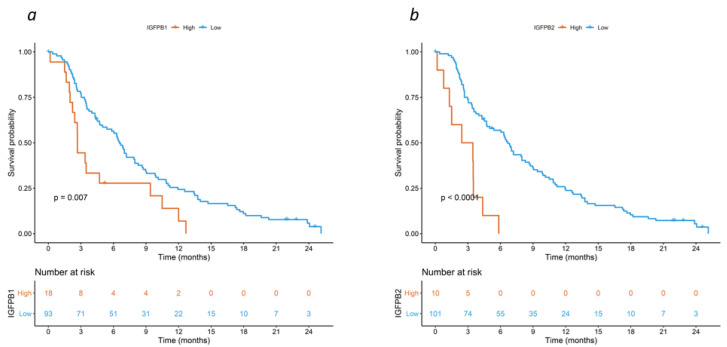
Kaplan–Meier curves for overall survival by expression of (**a**) IGFBP-1 and (**b**) IGFBP-2 in the validation cohort.

The median cytokine concentrations were 20.13 ng/mL for IGFBP-1 (interquartile range (IQR) 63.01), 696.51 ng/mL for IGFBP-2 (IQR 827.49), 8342 ng/mL for SAA (IQR 92,055.8), and 1.23 ng/mL for angiotensin II (IQR 0.62) (Supplementary Files, Table S2), with only a weak (if any) correlation between the four cytokines (Supplementary Files, Figure S3). High concentrations (i.e., higher than the predefined cut-off value) were found in 18 (16.2%), 10 (9%), 51 (45.9%) and 1 (0.9%) patients, respectively. In univariable analysis, IGFBP-1 (HR 2.04 [95%CI: 1.20–3.45], FDR = 0.024), IGFBP-2 (HR 4.17 [95%CI: 2.08–8.33], FDR = 0.001), and serum amyloid A (SAA) (HR 2.13 [95%CI: 1.43–3.23], FDR = 0.001) were significant prognostic factors. Patients with high concentrations of IGFBP-1 (2.7 vs. 6.6 months), IGFBP-2 (2.9 vs. 6.7 months), and SAA (3.5 vs. 8.7 months), had a shorter median OS (Figure 2, and Figure S4 in the Supplementary Files). After adjusting for ALP and WB-MATV, the association with OS was confirmed for IGFBP-1 (HR 2.70 [95%CI: 1.56–4.76], FDR = 0.006) and IGFBP-2 (HR 3.33 [95%CI: 1.61–6.67], FDR = 0.008), while a trend was observed for SAA (HR 1.54 [95%CI: 0.99–2.44], FDR = 0.092) (Supplementary Files, Table S3).

**Figure 3:**
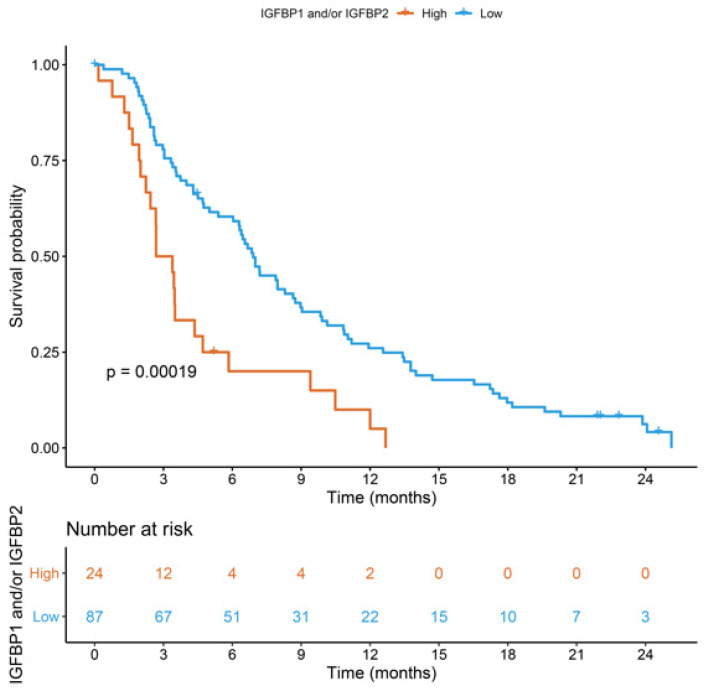
Kaplan–Meier curves for overall survival by combined expression of IGFBP-1 and IGFBP-2 in the validation cohort.

High concentrations of IGFBP-1 and/or IGFBP-2 were detected in 24 (21.6%) patients. These had a worse OS than those with low concentrations of both IGFBP-1 and IGFBP-2 (3.0 vs. 6.9 months, HR 2.44 [95%CI: 1.52–4.0], FDR = 0.002). Patients’ characteristics in terms of the combined expression of IGFBP1 and IGFBP2 are reported in Table S4 (Supplementary Files). In a multivariable analysis including ALP and WB-MATV, the independent prognostic role of the combined assessment of IGFBP-1 and IGFBP-2 was confirmed (HR 3.03 [95%CI: 1.79–5.0], FDR < 0.001) (Figure 3).

## 4. Discussion

In this study, we have shown that the expression of circulating cytokines independently predicts the prognosis of chemorefractory CRC. By analyzing a large panel of cytokines, we have found that patients with high levels of IGFBP-1 and/or IGFBP-2 have a significantly worse outcome than those with low levels of both cytokines.

Notwithstanding the established role of circulating cytokines in a number of processes that promote and sustain cancer development and progression, data about the clinical utility of cytokine testing in solid tumors including CRC are very scarce [18,19]. As a result, there is no circulating cytokine that is routinely assessed in clinical practice to help prognostication, treatment selection or response prediction. This is especially notable if we consider the pressing need to better account for the inherent cancer biological heterogeneity and the overall potential of liquid biopsy in this setting. Furthermore, beyond their key biological functions, circulating cytokines share some ideal properties (i.e., easily and repeatedly accessible over time, short half-life), which make them optimal candidate biomarkers for clinical use [20,21].

Our study aimed to address this evidence gap. By taking advantage of plasma samples from patients enrolled in three clinical trials, we simultaneously tested 182 cytokines, which play a role in diverse physiological and pathological mechanisms such as, among others, tumorigenesis, cancer development and metastasis, cell viability/growth/proliferation and differentiation, cell death/apoptosis, extracellular matrix remodeling, fibrosis, adhesion, immunity, inflammation, angiogenesis, and glucose and lipid metabolism. Among these, only IGFBP-1 and IGFBP-2 were found to be independently associated with OS both in the discovery and validation cohort, while a non-statistically significant association was observed between SAA and prognosis. Confirmation in the validation cohort of a favorable prognostic effect of angiotensin II, as observed in the discovery cohort, could not be achieved due to only one patient having high level of this cytokine in the former.

The main concern when running multiple analyses and comparisons is the risk of false positive results. While our study is no exception to this rule, we did take effective measures to mitigate against such risk, including stringent criteria for statistical significance and internal validation. More importantly, the biological plausibility of our findings is supported by the similar function of the two abovementioned cytokines that, while not being strongly associated with each other in our series, belong to the same insulin-like growth factor family. This consists of two ligands (IGF-I and IGF-II), two tyrosine–kinase receptors (IGF-1R and IGF-2R), and six IGF-binding proteins (IGFBP-1 through IGFBP-6) [22]. The association between the IGF pathway and CRC has long been recognized [23]. The deregulation of this pathway can induce increased cell proliferation, the inhibition of apoptosis, the stimulation of angiogenesis, the promotion of tumor invasion/metastasis, and resistance to treatments such as anti-EGFR monoclonal antibodies [22,23,24,25,26]. It is interesting to note, however, that neither IGF-1 nor IGF-2 (both of which were included in our large cytokine panel for the discovery cohort alongside IGFBP-3) appeared to have any prognostic value. Overall, this would suggest a biological effect of the IGF binding proteins that is independent of their ligands. In line with this hypothesis, studies have previously shown that, in addition to changing the IGF ligand half-lives and modulating their interaction with the IGF receptors, IGFBP-1 and IGFBP-2 can directly promote cancer development by interacting with the α5/β1 integrin receptor or suppressing the expression of E-cadherin [27,28,29]. It is possible that this dual activity could also explain the inconsistent results from previous studies looking at the clinical value of IGFBP-1 and IGFBP-2, as well as their tumor- or stage-dependent prognostic effect [30,31,32,33,34,35,36,37,38,39]. Of course, this hypothesis would need to be confirmed in appropriately designed mechanistic studies.

Another possible concern regarding our findings could be their limited generalizability. While analyzing patients who were previously recruited in clinical trials allowed us to match cytokine expression with well-annotated clinical data, the risk exists that our study population may not be entirely representative of the general population of chemorefractory CRC patients. Furthermore, using a prospective series of patients treated with regorafenib as a validation cohort raises the question as to whether the observed association between IGFBP-1 and IGFBP-2 and survival is more reflective of a predictive rather than a prognostic effect. It should be noted, however, that our discovery cohort was substantially more heterogeneous, all the patients being treated according to standard practice including best supportive care for those deemed not suitable for further active treatment. There is no doubt that this is an exploratory study, and our results still need external validation in independent larger series. In this regard, the use of enzyme-linked immunoassays with pre-defined cut-offs after an initial screening by a multiplex assay, should increase the reproducibility of our analysis.

We acknowledge the limitations of our study. These include general points such as the exploratory nature, the relatively small sample size, which is reflected by the very small number of subjects at risk in the Kaplan–Meier curves, and the retrospective design, as well as more specific considerations. First, while the data suggest a prognostic effect of IGFBP-1 and IGFBP-2, they do not clarify the underlying biological mechanisms, nor do they provide any insight regarding how to best use this information for clinical practice. It is unclear, for instance, whether these poor-prognosis patients are likely to be treatment resistant and, therefore, optimal candidates for best supportive care with the intent to minimize unnecessary toxicities and preserve quality of life, or could rather benefit from further active therapy. Second, our findings are from a chemorefractory patient population, and it is unknown whether these may apply to other clinical contexts such as the treatment-naïve or peri-operative setting. Third, despite the abovementioned attempts to reduce the risk of false positive results, we cannot exclude the fact that the association between IGFBP-1, IGFBP-2 and outcome could be a random finding. Finally, even if 182 cytokines were analyzed in the discovery cohort, these are still limited and far from accounting for the overall number and complexity of cancer pathways influenced by these cell-signaling mediators.

In conclusion, this analysis showed that circulating cytokines may be clinically useful biomarkers for chemorefractory CRC, and should be given more attention by researchers in the field of liquid biopsy. Although our study has the largest panel of circulating cytokines ever conducted in this disease setting, the association between high levels of IGFBP-1 and/or IGFBP-2 and worse survival remains hypothesis-generating. The external validation of our findings in larger series is needed to confirm this hypothesis.

## Figures and Tables

**Table 1:** Patient characteristics in the discovery and validation cohorts.

	Discovery Cohort (N = 85)	Validation Cohort (N = 111)
Age		
Median (IQR)	64 (13)	66 (14)
Sex		
Male	48 (56.5%)	66 (59.5%)
Female	37 (43.5%)	45 (40.5%)
ECOG PS		
0	39 (45.9%)	51 (45.9%)
≥1	46 (54.1%)	59 (53.2%)
Unknown		1 (0.9%)
Primary tumor location		
Left	61 (71.8%)	74 (66.7%)
Right	21 (24.7%)	20 (18.0%)
Unknown	3 (3.5%)	17 (15.3%)
*RAS/BRAF* status		
*RAS/BRAF* mutated	42 (49.4%)	71 (64.0%)
*RAS/BRAF* wild-type	35 (41.2%)	38 (34.2%)
Unknown	8 (9.4%)	2 (1.8%)
N of metastatic sites		
1	15 (17.6%)	14 (12.6%)
≥2	70 (82.4%)	97 (87.4%)
Liver metastasis		
Present	58 (68.2%)	82 (73.9%)
Absent	27 (31.8%)	29 (26.1%)
Peritoneal metastasis		
Present	22 (25.9%)	21 (18.9%)
Absent	63 (74.1%)	90 (81.1%)
Baseline ALP (UI/L)		
Median (IQR)	140 (137)	168 (203)
Baseline CEA (μg/L)		
Median (IQR)	73.8 (417)	132 (286)
Time from diagnosis to blood sampling (months)		
Median (IQR)	31.3 (38)	36.3 (35)
N of prior lines of systemic therapy		
Median (IQR)	2 (1)	2 (1)
Systemic therapy after blood sampling		
Yes	64 (75.3%)	111 (100%)
No	21 (24.7%)	0 (0%)

Abbreviations: ALP, alkaline phosphatase; CEA, carcinoembryonic *antigen*; ECOG, Eastern Cooperative Oncology Group; IQR, interquartile range.

## Data Availability

Raw study data are available upon reasonable request.

## Supplementary Materials

The following supporting information can be downloaded at: https://www.mdpi.com/article/10.3390/cancers15245823/s1. Figure S1: Principal component analysis of cytokine expression by source study in the discovery cohort. Figure S2: Kaplan–Meier curve for overall survival by expression of (a) angiotensin II and (b) Serum Amyloid A in the discovery cohort. Figure S3: Correlation between the cytokines analyzed in the validation cohort (Spearman rank correlation). Figure S4: Kaplan–Meier curve for overall survival by expression of (a) angiotensin II and (b) Serum Amyloid A in the validation cohort Table S1: Dilution, range, and sensitivity for cytokines tested by ELISA in the validation cohort. Table S2: Median values of IGFBP1, IGFBP2, SAA and angiotensin II in patients from the validation cohort and healthy volunteers. Table S3: Univariable and multivariable analyses in the validation cohort. Table S4: Patient characteristics according to the expression of IGFBP-1 and/or IGFBP-2 in the validation cohort.

## Abbreviations

ALP	alkaline phosphatase
CI	confidence intervals
CRC	colorectal cancer
ECOG	Eastern Cooperative Oncology Group
ELISA	enzyme-linked immunosorbent assay
FDR	false discovery rate
HR	hazard ratio
IGFBP-1	insulin-like growth factor-binding protein 1
IGFBP-2	insulin-like growth factor-binding protein 2
OD	optical density
OS	overall survival
PS	performance status
SAA	serum amyloid A
WB-MATV	whole-body metabolically active tumor volume
