# Supplementary material for: Prognostic Value of Circulating Cytokines in Chemorefractory Colorectal Cancer
Source: Cancers (Basel). 2023 Dec 13;15(24):5823. doi: 10.3390/cancers15245823 (PMC10742027; doi:10.3390/cancers15245823)
Supplement: Supplementary file 1 [file cancers-15-05823-s001.zip › cancers-2718932-SI.pdf]

Supplementary files

Table S1. Dilution, range, and sensitivity for cytokines tested by ELISA in the validation cohort

	**Dilution**		**Range**	**Sensitivity**
	Healthy volunteers	Study patients		
IGFBP-1	100x	200x	31.2 pg/mL - 2000 pg/mL	1 pg/mL
IGFBP-2	100x	300x	8.23 pg/mL - 6000 pg/mL	20 pg/mL
SAA	300x	2000x	0.41 ng/mL - 300 ng/mL	0.5 ng/mL
ANG-II	no dilution		31.25 pg/mL - 2000 pg/m	18.75 pg/mL

Abbreviations: *ANG-II, Angiotensin II; IGFBP-1, insulin-like growth factor-binding protein 1; IGFBP-2, insulin-like growth factor-binding protein 2; SAA, Serum Amyloid A.*

Figure S1. Principal component analysis of cytokine expression by source study in the discovery cohort

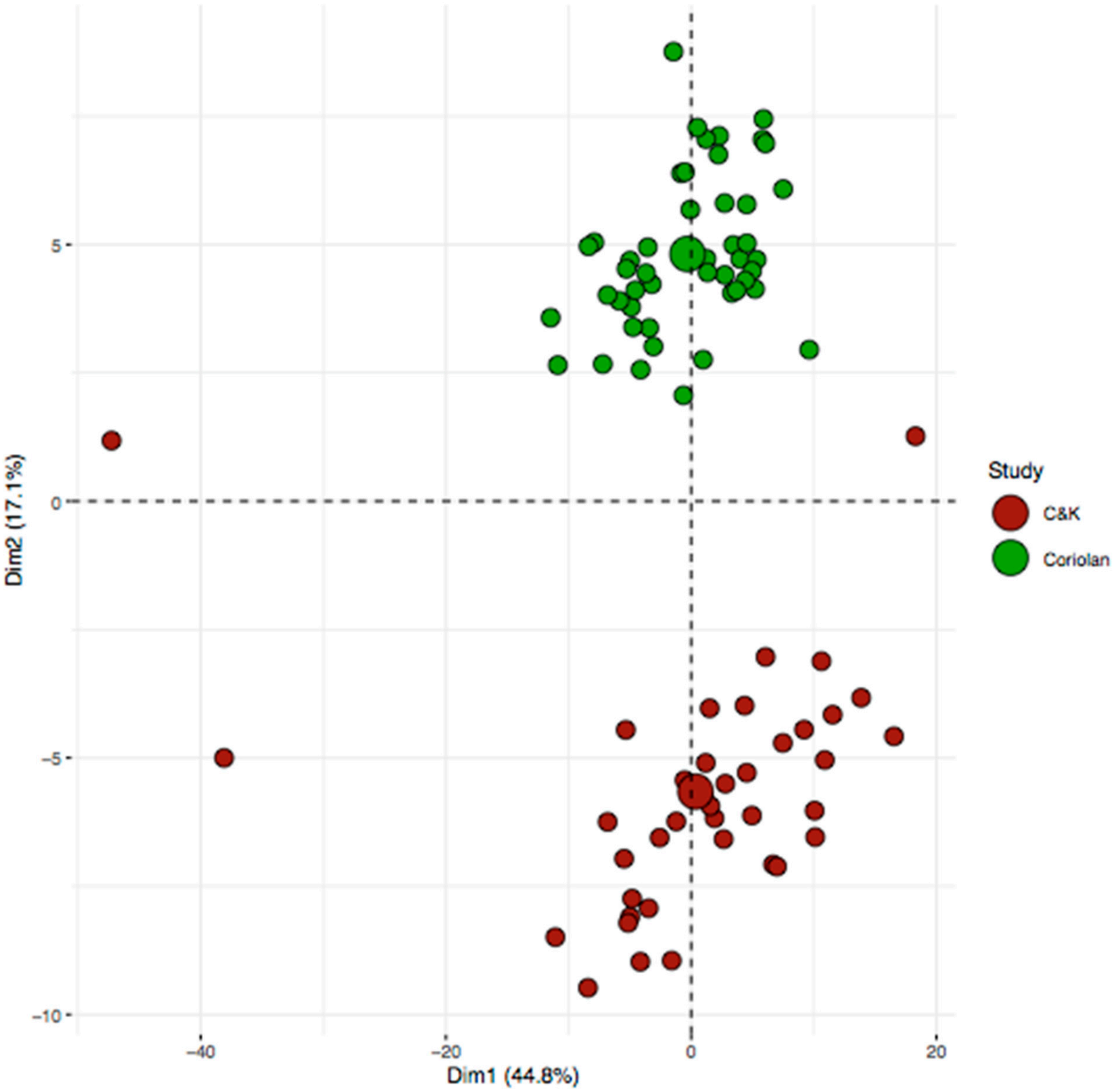

Abbreviations: *C&K, Chronos & Kairos.*

Figure S2. Kaplan-Meier curve for overall survival by expression of *(a)* Angiotensin II and *(b)* Serum Amyloid A and in the discovery cohort

a

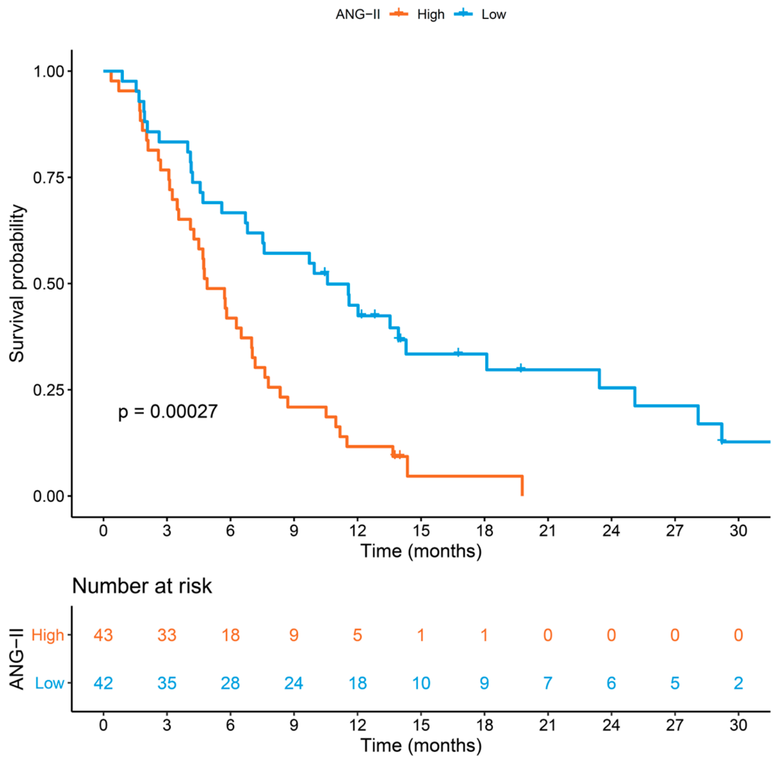

b

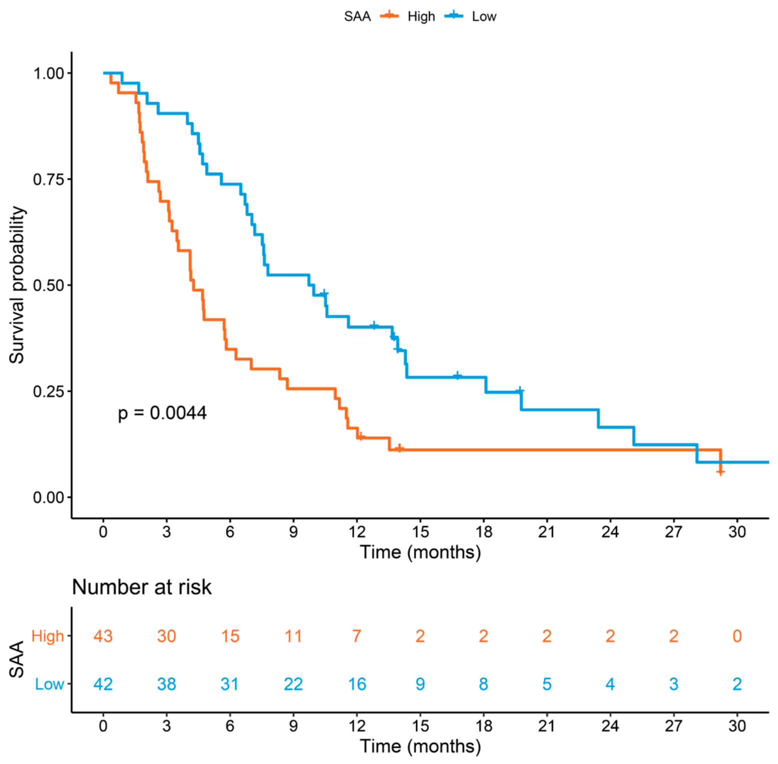

Abbreviations: *ANG-II, Angiotensin II; SAA, Serum Amyloid A.*

Table S2. Median values of IGFBP1, IGFBP2, SAA and angiotensin II in patients from the validation cohort and healthy volunteers

	**Validation cohort**	**Healthy volunteers**
IGFBP1 (pg/ml)<br>Median	42,821	33,292
IGFBP2 (pg/ml)<br>Median	696,509	508,958
SAA (ng/ml)<br>Median	8,418	1,962
Angiotensin II (pg/ml)<br>Median	703	921

Abbreviations: *ANG-II, Angiotensin II; IGFBP-1, insulin-like growth factor-binding protein 1; IGFBP-2, insulin-like growth factor-binding protein 2; SAA, Serum Amyloid A;*

Figure S3. Correlation between the cytokines analysed in the validation cohort (Spearman rank correlation)

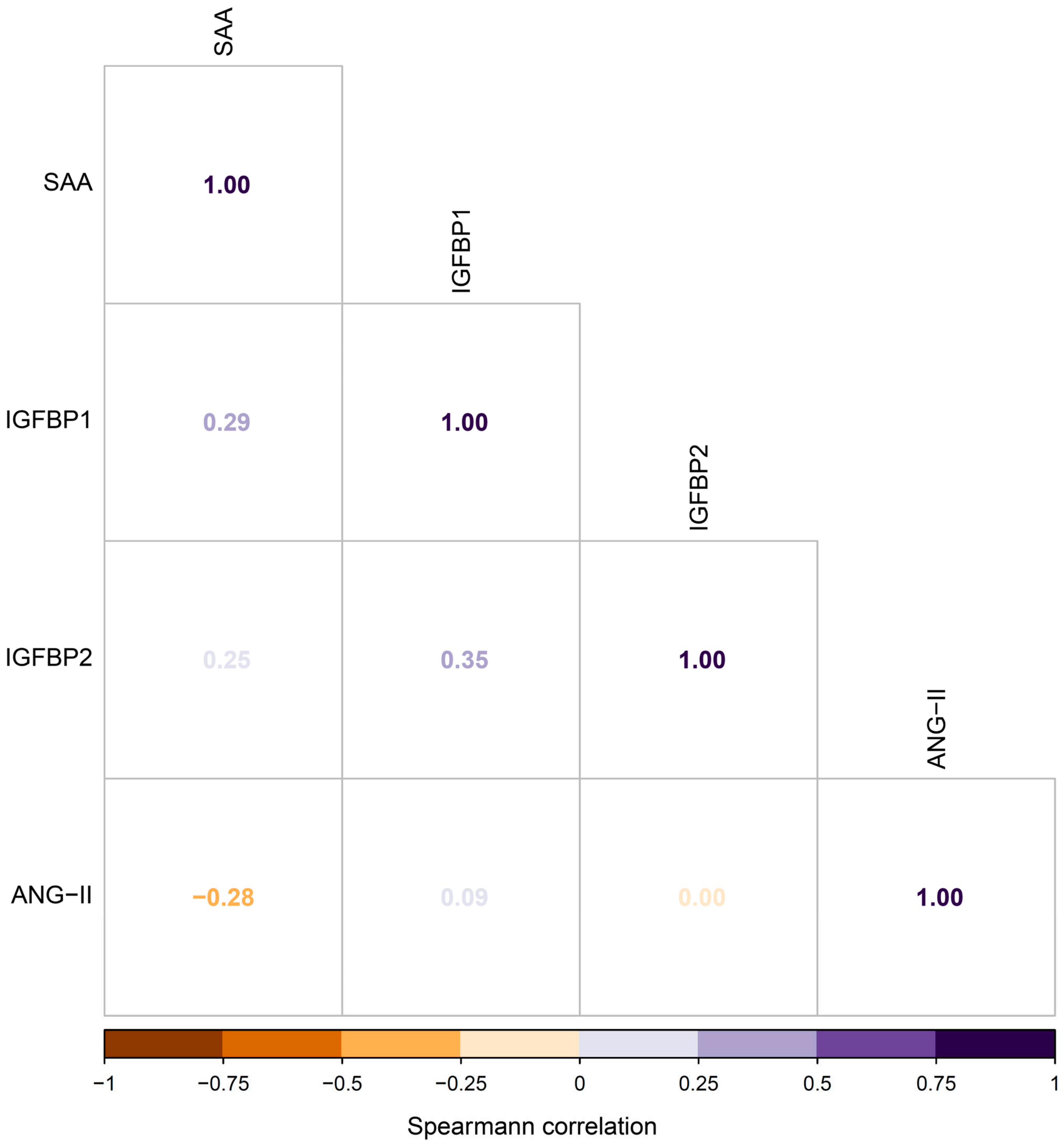

Figure S4. Kaplan-Meier curve for overall survival by expression of *(a)* Angiotensin II and *(b)* Serum Amyloid A and in the validation cohort

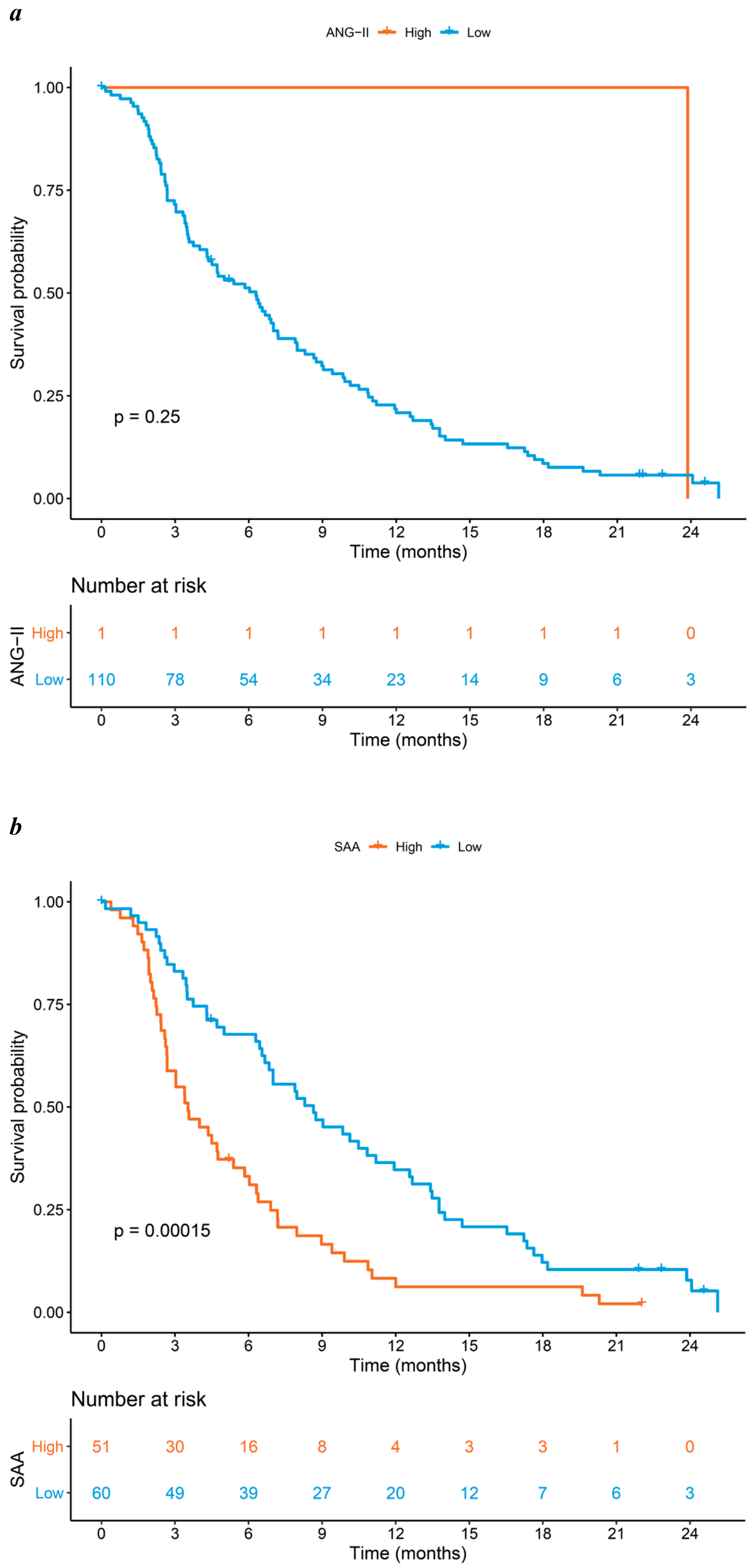

***Abbreviations:** ANG-II, Angiotensin II; SAA, Serum Amyloid A.*

Table S3. Univariable and multivariable analyses in the validation cohort

Cytokine	Univariable analysis		Multivariable analysis	
	HR (95% CI)	FDR	HR (95% CI)	FDR
IGFBP1	2.04 (1.20-3.45)	0.024	2.70 (1.56-4.76)	0.006
IGFBP2	4.17 (2.08-8.33)	0.001	3.33 (1.61-6.67)	0.008
SAA	2.13 (1.43-3.23)	0.001	1.54 (0.99-2.44)	0.092
IGFBP1 + IGFBP2	2.44 (1.52-4.0)	0.002	3.03 (1.79-5.0)	<0.001

Abbreviations: *ANG-II, Angiotensin II; IGFBP-1, insulin-like growth factor-binding protein 1; IGFBP-2, insulin-like growth factor-binding protein 2; SAA, Serum Amyloid A; FDR, false discovery rate; HR, hazard ratio; CI, confidence intervals.*

Table S4. Patient characteristics according to the expression of IGFBP-1 and/or IGFBP-2 in the validation cohort

	**IGFBP-1 and IGFBP-2 low expression (N=87)**	**IGFBP-1 and/or IGFBP-2 high expression (N=24)**
Age		
median (IQR)	66 (9)	67 (8)
Sex		
Male	53 (60.9%)	13 (54.2%)
female	34 (39.1%)	11 (45.8%)
ECOG PS		
0	44 (50.5%)	7 (29.2%)
≥1	43 (49.5%)	17 (70.8%)
Primary tumor location		
Left	61 (70.1%)	13 (54.2%)
Right	15 (17.2%)	5 (20.8%)
Unknown	11 (12.7%)	6 (25%)
RAS/BRAF status		
RAS/BRAF mutated	56 (64.3%)	15 (62.5%)
RAS/BRAF wild-type	30 (34.5%)	8 (33%)
Unknown	1 (1.2%)	1 (4.5%)
N of metastatic sites		
1	12 (13.8%)	2 (8.4%)
≥2	75 (86.2%)	22 (91.6%)
Liver metastasis		
Present	63 (72.4%)	19 (79.2%)
Absent	24 (27.6%)	5 (20.8%)
Peritoneal metastasis		
Present	15 (17.2%)	6 (25%)
Absent	72 (82.8%)	18 (75%)
Baseline ALP		
Median (IQR)	151 (189)	239 (308)
Baseline CEA		
Median (IQR)	117 (311)	137 (833)
Time from diagnosis to blood sampling		
median (IQR)	40 (36)	25 (36.9)

Abbreviations: *ALP, alkaline phosphatase; CEA, carcinoembryonic antigen; ECOG, Eastern Cooperative Oncology Group; IQR, interquartile range.*
